# Association between Swallowing Disorders and Independence during Mealtime in Neurological Diseases: A Cross-sectional Study

**DOI:** 10.1055/s-0046-1819715

**Published:** 2026-04-24

**Authors:** Valeria Crispiatico, Giulia Fusari, Cinzia Baldanzi, Chiara Vitali, Francesca Tedeschi, Claudia Bazzotti, Federico Favini, Davide Cattaneo

**Affiliations:** 1 Department of Psychology, Università degli Studi di Milano Bicocca, Milan, Italy; 2 Istituti di Ricovero e Cura a Carattere Scientifico Don Carlo Gnocchi, Fondazione Don Carlo Gnocchi – Organizzazione Non Lucrativa di Utilità Sociale (ONLUS), Milan, Italy; 3 Neurorehabilitation department, Fondazione Don Carlo Gnocchi – Organizzazione Non Lucrativa di Utilità Sociale (ONLUS), San Donato Milanese, Italy; 4 Department of Physical Medicine and Rehabilitation, L'Ospedale di Circolo - Fondazione Macchi, ASST Sette Laghi, Varese, Italy; 5 Department of Pathophysiology and Transplantation, Università degli Studi di Milano Statale, Milan, Italy

**Keywords:** oropharyngeal dysphagia, mealtime, independence, neurological diseases, deglutition disorders

## Abstract

**Introduction:**

Mealtimes are important for patient's autonomy and independence. They are linked to values and are a source of normality and fulfilment. Independence during mealtimes means eating and drinking safely and independently. However, the presence of oropharyngeal dysphagia (OD) can compromise independence, particularly among patients with neurological conditions.

**Objective:**

The present cross-sectional study aims to investigate the association between functional independence during meal and multidimensional assessment of neurological subjects with OD.

**Methods:**

Subjects with OD (DOSS score < 6) and diagnosis of multiple sclerosis, Parkinson's disease, or stroke were recruited. Demographic and clinical data were collected. The swallowing assessment included the Penetration Aspiration Scale (PAS) and the Dysphagia Outcome and Severity Scales (DOSS) during an instrumented assessment, self-reported questionnaire (Eating Assessment Tool-10 - EAT-10), Body Mass Index, a meal observation scored with the Mealtime Assessment Scale (MAS) and cognitive assessment using the Mini-Mental State Examination. The level of independence was assessed using the American Speech-Language-Hearing Association National Outcomes Measurement System (ASHA NOMS) Swallowing Scale. Univariate and multivariate logistic models were used to analyze data.

**Results:**

Fifty-eight subjects were recruited (18 MS, 20 PD and 20 stroke), among whom 41 (70%) were defined as dependent during mealtime. In the multivariate analysis, EAT-10 score (odds ratio [OR] [95%CI] = 3.25 [1.64–11.08]), diseases duration (OR [ 95%CI] = 0.87 [0.74–0.97]) and MAS safety (OR [ 95%CI] = 1.26 [1.06–1.65]) were significantly associated with independence during mealtime.

**Conclusion:**

Subject's performance during meal, self-reported questionnaire, and diseases duration are independently associated with independence. The present study broadens the focus on dysphagia, underling the importance of identifying all variables able to increase subjects' independence and autonomy at mealtime.

## Introduction

Mealtime is closely associated with a multitude of values and customs, and for patients, it has become an essential part of maintaining “a sense of normality”. 1 This fundamental aspect of life facilitates the fulfilment of biological (e.g., nutrition and hydration) and psychosocial needs. It also signifies a pivotal juncture in daily life where patients' autonomy and independence can be cultivated. 2 The term “independence during mealtimes” can be defined as the capacity to consume food and beverages in a manner deemed to be both safe and autonomous, entailing the occasional use of self-cueing. This concept could be regarded as a pivotal aspect of person-centered mealtime practices. 3 4 Moreover, the absence of independence during mealtime appeared to be a predictor of aspiration pneumonia in elderly subjects. 5 6 The presence of oropharyngeal dysphagia (OD), a functional impairment that either prevents or limits the intake of food and fluids, and which makes swallowing unsafe, inefficient, uncomfortable, or affects quality of life, 7 has the potential to influence individuals' experiences during mealtimes. Oropharyngeal dysphagia is a prevalent symptom of numerous neurological disorders, with the most notable examples being stroke (prevalence: 8.1–80%), Parkinson's disease (PD; 11–81%), 8 and multiple sclerosis (MS; 43%). 9 While the majority of neurological patients retain the capacity to feed orally without significant complications, the implementation of compensatory strategies is often essential to ensure optimal safety during the act of swallowing. 10 11 These strategies encompassed behavioral and social recommendations (e.g., avoiding noisy environments, implementing postural adjustments, and receiving supervision from caregivers) and dietary restrictions (e.g., alterations in rheology, viscosity, volume, and consistency of food or liquids). 10 11 Consequently, a distinctive challenge arises in aligning person-centered mealtime practices with eating-related safety needs, 2 and the independence during mealtime could be affected.

To date, several studies have looked at Speech Language Therapy (SLT) dysphagia-management practices, 12 but little information is available on how independence during meal is affected by OD.

Oropharyngeal dysphagia is a multidimensional phenomenon, 7 13 and, as such, the multidisciplinary dysphagia team must examine a range of factors using a complete multidimensional assessment, 7 13 including:

(1) instrumental assessment (videofluoroscopy [VFS] or fiberoptic endoscopic evaluation of swallowing [FEES]) to investigate the safety and the efficiency of the swallowing function; 13
(2) A clinical assessment is conducted to evaluate the structures and functions of the oral and pharyngeal musculature. Additionally, observations of food or fluid swallowing trials are made to gain insight into the underlying pathophysiological mechanisms of dysphagia; 13
(3) self-evaluation questionnaires of swallowing verifying the impact of OD on the subject's daily life activities; 13
(4) assessment of the subject's performance during the meal to verify the influence of personal and environmental factors on functional independence during the meal; 14
(5) nutritional status to verify the presence of malnutrition; 15
(6) a global assessment of cognitive skills evaluating subjects' ability to retain and autonomously apply compensatory strategies. 13 Moreover, different studies support the intersection in the neural network between swallowing behavior and cognitive function. 16

Although many studies have correlated these different factors related to OD to each other with controversial results, 14 17 18 no studies have explored the relationship between these variables and independence during meals.

Therefore, our study aims to investigate the association between functional independence during meals and OD assessments. Indeed, identifying the components of OD most affecting independence during meals can provide clinically relevant information to plan a rehabilitative intervention tailored to the specific needs of each subject.

## Methods

In the present cross-sectional study, a convenience sample of subjects with OD affected by stroke, PD, or MS were recruited among inpatient and outpatient subjects of Don Gnocchi Fondation between January 2021 and June 2022.

The inclusion criteria were: 1) diagnosis of stroke, PD, or MS; 2) age between 18 and 90 years; 3) understanding of the Italian language; 4) full oral nutrition; and 5) subjects with swallowing impairment (i.e., Dysphagia Outcome and Severity Scale < 6) 19 assessed by instrumental assessment.The exclusion criteria were: 1) other concomitant neurological diseases; 2) history of head and neck cancer; 3) the presence of vigilance disorders.

The study's procedures were approved by the institutional ethical committee of Don Gnocchi Fondation. All procedures performed in the study followed the Declaration of Helsinki and its later amendments or comparable ethical standards. All subjects provided written informed consent to study participation.

## Clinical Assessment

### Primary Outcome

The primary outcome was the functional independence during meals, which was measured using the American Speech-Language-Hearing Association National Outcomes Measurement System (ASHA NOMS) Scale Swallowing. 20 The ASHA NOMS is a 7-point outcome scale that ranges from the least functional level 1 to the most functional level 7. It rates severity based on the subject's ability to meet nutritional needs and independence with compensatory strategies, containing different information regarding the feeding modality, the nutrition and supervision level, cueing etc.

Although the scale is multifactorial, the rater based the score solely on their assessment of the patient's autonomy and need for cues.

### Clinical and Instrumental Associated Factors

The following clinical and demographic data were collected for each subject: age, gender, diagnosis, disease duration, presence of comorbidities (i.e., dysarthria, dysphonia and aphasia), and disease severity at Barthel Scale. 21

### Swallowing Safety and Efficacy

Based on the instrumental assessment, the Penetration Aspiration Scale (PAS) 22 and the Dysphagia Outcome and Severity Scale (DOSS) 19 were completed. The PAS 22 is an ordinal scale including scores from 1 to 8, with scores 1 to 2 considered to reflect normal swallowing function, while scores 6 to 8 reflect tracheal aspiration.

The DOSS 19 is a straightforward, user friendly, 7-point scale (ranging from 1, indicating severe dysphagia, to 7, indicating normal swallowing ability) that has been developed to provide a systematic assessment of the functional severity of dysphagia based on objective criteria. This assessment enables the formulation of recommendations regarding the appropriate diet, level of independence, and type of nutrition.

### Mealtime Assessment

The mealtime observation was evaluated using the Mealtime Assessment Scale (MAS). 14 The scale comprises 26 items and is divided into 4 subscales. The structures, functions, and activities that impact upon the act of eating, the environmental factors that influence this act, and the safety and efficacy of swallowing during the act of eating. To quantify the level of impairment in each aspect, a percentage can be calculated for both the safety and efficacy sections. A higher score or percentage indicates a greater degree of unsafe or ineffective swallowing during the meal. Furthermore, the time required for the subject to complete the meal is documented.

### Self-reported Questionnaire of Dysphagia-related Symptom

The Eating Assessment Tool (EAT-10) 23 is a symptom-specific validated questionnaire including 10 questions. It is scored from 0 - “no problem” to 4 - “severe problem,” regarding the severity of symptoms of OD. The cut-off score of ≥ 3 suggests a potential swallowing problem.

### Nutritional Status

Subject's weight and height were recorded, and Body Mass Index (BMI) was calculated as a measure of nutritional status. According to the consensus-based criteria proposed by European Society for Clinical Nutrition and Metabolism (ESPEN), a BMI ≤ 18.5 kg/m ^2^ suggests of a condition of malnutrition. 24

### Cognitive Assessment

Cognitive function was evaluated using the Mini-Mental State Examination (MMSE) test as a screening procedure. 25 The test comprises 30 items pertaining to diverse cognitive domains, enabling an estimation of the severity and progression of cognitive impairment. The total score can range between 0 and 30, with the following interpretations: a score < 18 indicates moderate-to-severe cognitive impairment; a score of 18 to 24 indicates mild cognitive impairment; and a score ≥ 25 indicates normal cognitive status. The total score was adjusted for age and education level.

The dependent variables (ASHA NOMS-Swallowing scale) were compiled by the same evaluator for all patients and were done so blindly. The other independent variables were assessed and collected by 3 speech therapists with a minimum of 5 years' experience in the management of swallowing disorders.

### Data Analysis

Data are reported as means and standard deviations, medians and ranges, or absolute and relative frequencies when appropriate. The Kruskal-Wallis (KW) test was used to compare the three diseases at baseline.

First, subjects with an ASHA total score ≥ 6 were categorized as “independent in eating and drinking.” Then, univariate logistic regression was used to assess association between dependence in eating and drinking (dependent variable) and outcomes collected during the multicomponent swallowing assessment (independent variables). Other independent variables included age, gender, diagnosis, disease duration, and disease severity according to the Barthel Scale. 24 Likewise, multivariate logistic models were used to assess multiple associations between the dependent and independent variables. The statistically significant variables identified in the univariate analysis were incorporated into a multivariate logistic regression model using the R stepwise procedure (step.aic function in the R “MASS” package) to derive a parsimonious model. The results included the β coefficients, McFadden coefficient of determination, residual standard error, and overall F-value. Residual plots were employed to ascertain whether the model assumptions were valid, while Cook's distance was utilized to evaluate the potential influence of outlying data points.

Significance was set at *p* < 0.05. Despite the implementation of a pairwise exclusion of missing data analysis approach, the dataset was deemed complete and devoid of any missing values.

## Result

Overall, we recruited a balanced sample of 58 subjects ( Table 1 ) with PD ( *N* = 20, 34%), stroke ( *N* = 20, 34%), and MS ( *N* = 18, 32%) showing a moderate disease severity as per the Barthel Scale score and high variability in disease duration. We also found a statistically significant difference in disease duration among the 3 populations (KW; *p* < 0.001), with subjects with stroke having a shorter disease duration.

**Table 1:** Demographic and clinical characteristics of study participants

Variable	Overall ( *n* = 58)	PD ( *n* = 20)	MS ( *n* = 18)	ST ( *n* = 20)
**Gender**				
*Female*	23 (39.7%)	7 (35.0%)	9 (50%)	7 (35.0%)
*Male*	35 (60.3%)	13 (65.0%)	9 (50%)	13 (65.0%)
**Age** (years)	48.3 (13.0)	43.6 (8.2)	56.1 (10.6)	45.7 (15.7)
**BMI** (malnutrition ≤ 18.5 kg/m ^2^ )	24.7 (3.6)	24.6 (3.3)	26.0 (3.8)	23.3 (3.4)
**Barthel Scale** (points – Best 0; Worst 100)	52.8 (27.8)	64.8 (26.4)	47.4 (25.9)	44.9 (27.9)
**Disease duration** (years)	11.8 (10.7)	11.6 (6.4)	22.4 (9.6)	2.4 (4.5)
**Comorbidities**				
*Pulmonary infections*	3 (5.2%)	1 (5.0%)	0 (%)	2 (10.0%)
*Loss of weight*	13 (22.4%)	4 (20.0%)	2 (11.1%)	7 (35.0%)
*Aphasia*	9 (15.5%)	0 (0%)	0 (0%)	9 (45.0%)
*Dysarthria*	26 (44.8%)	10 (50.0%)	8 (44.4%)	8 (40.0%)
*Dysphonia*	33 (56.9%)	13 (65.5%)	13 (72.2%)	7 (35.0%)
**ASHA NOMS**				
*Independent (total score ≥ 6)*	17 (29.3%)	7 (35.0%)	6 (33.3%)	4 (20.0%)
*Dependent (total score < 6)*	41 (70.7%)	13 (65.0%)	12 (66.7%)	16 (80.0%)
**Mealtime Assessment Scale**				
*Safety score (points)*	3.1 (1.6)	3.1 (1.5)	2.7 (1.7)	3.4 (1.6)
*Validity score (points)*	3 (2.1)	2.6 (1.4)	2.6 (2.0)	3.8 (2.7)
*Time of feeding (minutes)*	23.7 (6.9)	24 (5.8)	18.8 (5.5)	27.3 (7.0)
**EAT-10** (points)	9.4 (9.6)	5.9 (7.0)	9.4 (7.1)	13 (12.5)
**Penetration Aspiration Scale** (points)				
*Liquid*	3.0 (2.8)	2.5 (2.5)	2.1 (2.4)	4.3 (3.2)
*Half-solid*	1.3 (1.2)	1.2 (0.37)	1.1 (0.2)	1.8 (1.9)
*Solid*	1.3 (0.9)	1.1 (0.31)	1.1 (0.3)	1.6 (1.6)
**Dysphagia Outcome Severity Scale** (points)	4.8 (1.3)	5.3 (0.9)	5.2 (0.9)	3.9 (1.6)
**MMSE** (points)	25.4 (3.8)	25.1 (4.7)	26.2 (2.8)	24.9 (3.9)

**Abbreviations** : BMI, body mass index; EAT-10, Eating Assessment Tool-10; MMSE, Mini-Mental State Examination; MS, multiple sclerosis; PD, Parkinson disease; ST, stroke.

**Notes** : values are presented as n (%) or as mean (standard deviation). Demographic and clinical variables were compared among the three diagnoses using the Chi-squared test for categorical variables and the Kruskal-Wallis test with post hoc test and Bonferroni correction for continuous variables.

Based on the mean ASHA NOMS score, most subjects ( *N* = 41, 70%) were defined as dependent regarding eating and drinking (see Table 1 ). The instrumental assessment showed mild swallowing impairments (DOSS score in the whole sample: 4.8 [1.3]), with clinical assessment reporting normal nutritional status (BMI score in the whole sample: 24.7 [3.6]).

The results of uni-multivariate analyses are reported in Tables 2 , 3 . At the univariate level ( Table 2 ), several factors were found to be significantly associated with dependence during meal, including disease duration, dysarthria, EAT-10 score, MMSE score, and MAS safety and validity. The results of the univariate analyses were corroborated by the multivariate results ( Table 3 ), showing that disease duration (odds ratio [OR] [ 95%CI] = 0.87 [0.74–0.97]; *p* = 0.03), EAT-10 score (OR [ 95%CI] = 3.25[1.64–11.08]; *p* = 0.01), MAS safety (OR [ 95%CI] = 1.26 [1.06–1.65]; *p* = 0.01) was found to be associated with the dependency during meal.

**Table 2:** Demographic, clinical, and swallowing factors associated with independence during meal based on univariate analysis

Factors	Univariate analysis N = 58	
	OR (95%CI)	*p*
**Gender**	0.96 (0.91–1.01)	0.11
**Disease**	0.86 (0.21–5.93)	0.21
**Age** (years)	0.96 (0.91–1.01)	0.11
**BMI score** (malnutrition ≤ 18.5)	0.96 (0.80–1.27)	0.60
**Barthel Scale** (points)	0.98 (0.96–1.00)	0.17
**Disease duration (years)**	0.96 (0.91–1.01)	0.14
**Comorbidities:**		
*Pulmonary infections*	0.79 (0.07–19.12)	0.85
*Loss of weight*	2.66 (0.59–19.52)	0.26
*Aphasia*	0.79 (0.17–4.31)	0.77
*Dysarthria*	2.57 (0.76–9.71)	0.15
*Dysphonia*	1.79 (0.53–6.05)	0.35
**Mealtime Assessment Scale**		
*Safety score (points)*	2.56 (1.58–4.82)	0.001*
*Validity score (points)*	2.18 (1.39–3.90)	0.004*
*Time of feeding (minutes)*	1.12 (1.02–1.27)	0.04*
**EAT-10** (points)	1.17 (1.05–1.39)	0.03*
**Penetration Aspiration Scale**		
*Liquid*	NA	
*Half-solid*	1.99 (0.67–33.49)	0.42
*Solid*	1.76 (0.73–27.51)	0.45
**Dysphagia Outcome Severity Scale** (points)	0.29 (0.01–2.08)	0.39
**MMSE** (points)	0.85 (0.64–1.05)	0.19

**Abbreviations** : BMI, body mass index; EAT-10, Eating Assessment Tool-10; MMSE, Mini-Mental State Examination; NA, the model did not converge; OR, odds ratio.

**Notes** : for categorical variables, OR > 1 means a higher probability of the reference group than the control group to be independent during meal.

For continuous variables, OR > 1 means that an increase of 1 point in the value of the variable increases the probability of independence at mealtime.

*Significant *p* -values.

**Table 3:** Multivariate logistic regression analysis of clinical factors associated with independence during meal

Factors	Multivariate analysis	
	OR (95%CI)	*p*
**Disease duration** (years)	0.87 (0.74–0.97)	0.03*
**Mealtime Assessment Scale**		
*Safety score (points)*	3.52 (1.64–11.08)	0.01*
**EAT-10** (points)	1.26 (1.06–1.65)	0.03*

**Abbreviations** : EAT-10, Eating Assessment Tool-10; OR, odds ratio.

**Note** : *Statistically significant ( *p* < 0.05).

## Discussion

The aim of the present study was to investigate the relationship between swallowing assessment factors and independence during mealtimes in people with three different neurological diseases. The results showed that only 30% of the subjects were assessed as independent in terms of drinking and eating during mealtimes (ASHA NOMS Swallowing Scale ≥ 6).

The following paragraphs discuss the independent factors significantly associated with loss of independence during mealtimes in the multivariate model: swallowing safety during mealtimes (MAS safety score), self-perception (EAT-10 score), and disease duration.

### Association between MAS Safety Score and ASHA NOMS Level

The MAS safety score, defined as the “[…] ability to ingest all needed calories and water with no respiratory complications” 14 , was identified as an independent factor associated with independence during mealtimes. The results suggested that subjects experiencing impaired independence during mealtimes also exhibited concurrent safety impairments. This finding is consistent with the extant literature suggesting that a lack of dependence on eating is significantly associated with the development of pneumonia. 5 6

While the safety of the meal was found to be a significant independent factor, no statistically significant correlations were found between ASHA NOMS, PAS, or DOSS scores, which exclusively evaluate the swallowing mechanism. The resultant finding may be attributable to several factors. The MAS safety score has been conceptualized as a tool designed for the evaluation of swallowing ability within a functional context. This tool takes into consideration the impact of cognitive, motor, and behavioral factors on safety. 14 During mealtimes, subjects must allocate attentional resources to eating over a prolonged period, even in the presence of distracting factors. During the instrumental assessment, subjects are required to focus exclusively on the act of swallowing. Second, it has been demonstrated that subjects require a greater quantity of food during meals than during instrumental examinations. 15

Consequently, mealtime assessment emerges as a more appropriate method for measuring functional and ecological outcomes, such as independence during meals. While the result may not be considered unexpected, it is nevertheless important to emphasize the significance of incorporating mealtime assessments into the management of dysphagia. This is because effective management of eating and drinking extends beyond the mere establishment of a functional swallow. 26

### Association between EAT-10 Score and ASHA NOMS Level

Swallowing self-perception (EAT-10 questionnaire) was another factor associated with independence during meals (ASHA NOMS), with individuals who perceive a greater impact of dysphagia on their functional health also exhibiting a higher degree of dependency during mealtimes. The current study demonstrated that the EAT-10 yielded valuable insights into the subjective impact of dysphagia, extending beyond the mere physical aspects of swallowing. It was found that a reduction in meal independence was perceived to be a more substantial impediment. To date, it has been well documented that clinical and instrumented assessments provide partial reflection of subjects' views on dysphagia symptoms, especially in subjects with neurological diseases. 27 28 29 Conversely, patient-reported measures have been shown to play an important role in patient-centered healthcare. Indeed, such measures have been demonstrated to improve communication, patient engagement and self-efficacy, given that patients may be more involved in goal setting. 13

It is also true that certain characteristics of the sample may have had some influence on the observed association, including age (i.e., a sample consisting primarily of younger individuals) and normal cognitive profile. Indeed, cognitive and neurosensory deficits associated with most neurological diseases play a key role in reducing awareness of swallowing impairments. 28 29 Furthermore, older subjects appear to demonstrate a reduced capacity for self-reporting dysphagic symptoms in comparison to younger individuals. 29 30

Furthermore, most subjects exhibited mild swallowing impairments, as evidenced by low PAS and high DOSS scores. This ceiling effect may have served to reduce the magnitude of the observed associations.

Consequently, for young individuals who are cognitively preserved and have moderate dysphagia, the effect that dysphagia has on their health status is associated with the degree of independence at mealtimes. This suggests that this should be an outcome to consider during multidisciplinary care.

### Association between Disease Duration and ASHA NOMS Level

Unexpectedly, disease duration was an independent factor associated with independence during meals, with shorter disease duration being associated with less independence. This result partially contrasts with the literature, according to which people with longer time from diagnosis have lower performance during meals and more severe swallowing impairments at the instrumental assessment. 27 We hypothesized that this difference could be influenced by the presence of three different diseases. Indeed, subjects with stroke had a statistically significant shorter disease duration than the two other neurological diseases (PD and MS) in the present sample. We suppose that stroke subjects could require stricter compensatory strategies, reducing independence during meals, especially in the first period post-event. In keeping with this hypothesis, a larger study by Braun et al. 31 evaluating dysphagia management in over 200 patients with neurologic diseases based on FEES found an association between restriction of oral intake and stroke diagnosis. Furthermore, the study of Tye et al. 27 on a sample of 178 subjects with neurodegenerative disorders (especially motor neuron disease and PD) having a longer disease duration showed that most of those subjects were able to maintain independence during mealtime and to be managed with conservative self-directed modifications.

Notwithstanding, it is imperative to exercise caution when interpreting these findings, particularly in light of the cross-sectional nature of the study and the modest sample size. The results of the current study would be able to be corroborated by a future longitudinal study utilizing a larger sample size. Furthermore, it would be worthwhile to include patients suffering from chronic stroke.

In conclusion, the present study represents the first in-depth assessment of the relationship between independence during mealtimes and a comprehensive multidimensional assessment of swallowing function in patients with neurological diseases. The present study's findings are in line with the prevailing hypothesis that a reduction in independence during mealtimes is concomitant with compromised safety during mealtimes and an increasing self-perception of the repercussions of dysphagia on functional health. Although instrumental assessment is generally considered to be the gold standard for diagnosing dysphagia, 13 no correlation was found between instrumental assessment scores and the patients' level of independence during mealtimes. This underscores the necessity for a more comprehensive evaluation of dysphagia, particularly with respect to the intricacy of the subjects assessed, to contemplate more efficacious functional outcomes. 5 6 14 27 Furthermore, the study found an inverse correlation between autonomy at mealtimes and disease duration. The findings are somewhat surprising and may be subject to bias due to the inclusion of three distinct population groups and the relatively small sample size.

It should be noted, however, that the present study was not without its limitations. First, the cross-sectional nature of the study precludes the establishment of casual relationships. Additionally, the heterogeneity of participants at this single institution may limit the generalizability of the findings.

Furthermore, most subjects in our sample exhibited mild swallowing and cognitive impairments, while their BMI remained uncompromised. Moreover, it is recognized that BMI is an unsatisfactory metric for estimating the risk of malnutrition. 32

Finally, the recommendations set out in the context of the coronavirus disease 2019 (COVID-19) pandemic have resulted in difficulties in the implementation of a well-standardized protocol which is commonly utilized in clinical practice for the bedside examination.

These limitations indicate potential avenues for future research. To enhance the external validity of the findings, a multicenter longitudinal trial is recommended to ascertain the correlation between independence and a multidimensional assessment of swallowing.

It is therefore recommended that subsequent studies include a greater number of participants experiencing more severe swallowing and cognitive impairments.

## Conclusion

Meals are an integral component of everyday life. Individuals afflicted with dysphagia encounter considerable limitations that have a detrimental effect on their quality of life. From a patient-centered perspective, the ability to eat independently and safely is considered a key outcome for the multidisciplinary rehabilitation team. The present study demonstrates that more than 70% of individuals diagnosed with dysphagia experience a lack of independence during mealtimes. The absence of independence appears to be associated not so much with impaired swallowing effectiveness and safety at the instrumental assessment, but rather with a lack of safety during mealtimes and an increased perception of the impact of swallowing disorders on one's health. It has been observed that a more recent onset of the disorder appears to be associated with a reduction in independence.

Consequently, the evaluation of dysphagia should encompass a comprehensive approach, extending beyond conventional clinical and instrumental swallowing assessments. This entails a meticulous examination of the entire meal process, encompassing direct observation of the meal, assessment of the environment, tools utilized, posture adopted, and the necessity for assistance. Additionally, an in-depth investigation into the patient's perception of their disorder and autonomy is imperative.

Concurrently, rehabilitation objectives ought to be articulated with respect to autonomy and engagement in mealtime activities, extending beyond mere enhancement of swallowing efficacy.
